# Engine of Innovation in Hospital Pharmacy: Applications and Reflections of ChatGPT

**DOI:** 10.2196/51635

**Published:** 2024-10-04

**Authors:** Xingang Li, Heng Guo, Dandan Li, Yingming Zheng

**Affiliations:** 1 Department of Pharmacy, Beijing Friendship Hospital Capital Medical University Beijing China

**Keywords:** ChatGPT, hospital pharmacy, natural language processing, drug information, drug therapy, drug interaction, scientific research, innovation, pharmacy, quality, safety, pharmaceutical care, tool, medical care quality

## Abstract

Hospital pharmacy plays an important role in ensuring medical care quality and safety, especially in the area of drug information retrieval, therapy guidance, and drug-drug interaction management. ChatGPT is a powerful artificial intelligence language model that can generate natural-language texts. Here, we explored the applications and reflections of ChatGPT in hospital pharmacy, where it may enhance the quality and efficiency of pharmaceutical care. We also explored ChatGPT’s prospects in hospital pharmacy and discussed its working principle, diverse applications, and practical cases in daily operations and scientific research. Meanwhile, the challenges and limitations of ChatGPT, such as data privacy, ethical issues, bias and discrimination, and human oversight, are discussed. ChatGPT is a promising tool for hospital pharmacy, but it requires careful evaluation and validation before it can be integrated into clinical practice. Some suggestions for future research and development of ChatGPT in hospital pharmacy are provided.

## Introduction

Hospital pharmacists play an important role in ensuring the safe and effective use of medications in hospitals. However, pharmacists also face many challenges, such as the increasing complexity of patients’ medication regimens, the potential risks of medication errors and drug-drug interactions, the constant updating of pharmaceutical knowledge, and the growing volume of pharmaceutical literature [1-4]. Due to the large volume of medical-related literature, pharmacists spend a lot of time searching, analyzing, and applying relevant information in clinics. In addition, it takes a lot of effort to keep up with the latest information on new drugs, clinical guidelines, adverse drug reactions, and drug-drug interactions. Therefore, how to quickly and effectively obtain and interpret medication-related information has emerged as a challenge for pharmacists.

With progress in deep learning and natural language processing technologies, generative large language models like ChatGPT have come to the forefront. Its outstanding performance in understanding context and generating text lays a good foundation for its wide application in various fields [5-9]. If ChatGPT is applied to hospital pharmacy, it may become a new tool for hospital pharmacists to solve the aforementioned problems and challenges, improve work efficiency, ensure safe and rational use of drugs, and even greatly change the status quo of hospital pharmacy operations and research [10].

This article aims to explore the application potential of ChatGPT in daily hospital pharmacy operations and research, and analyze the challenges and problems faced by ChatGPT. Simultaneously, we provide our suggestions for the development of artificial intelligence (AI) in this field.

## Overview of ChatGPT

**Figure 1:**
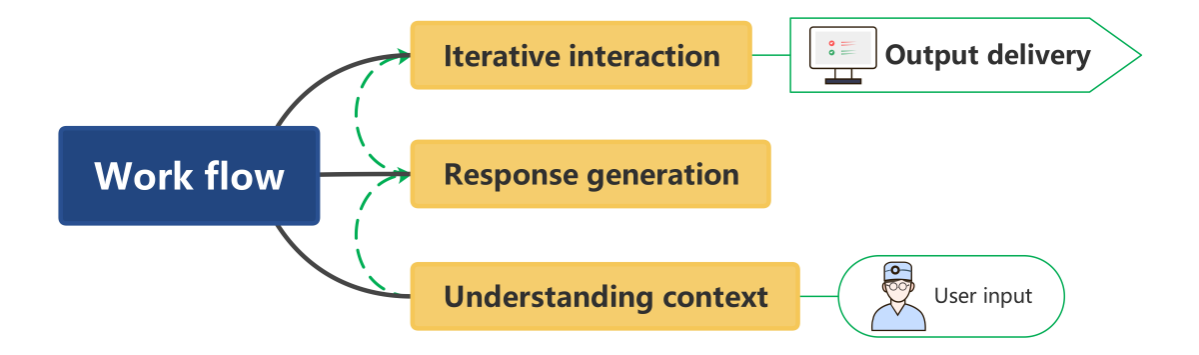
ChatGPT’s working principle of generating text.

The ChatGPT system is built upon a generative pretrained transformer (GPT) model, which has advantages in natural language processing (NLP). GPT pretrains a large amount of text data, such as books, articles, website content, etc, from which it acquires the ability to understand natural language deeply. The transformer architecture within GPT captures long-distance dependencies and contextual relationships in text through a self-attention mechanism, which enables ChatGPT to generate coherent and context-relevant responses, outputting content similar to human-written content. The working principle underlying its generation of text can be summarized as follows (Figure 1):

1. User input: ChatGPT receives user input information in text form, including single sentences and longer dialogue contexts.
2. Understanding context: ChatGPT processes the input text and uses pretrained parameters to understand the context and extract relevant information.
3. Response generation: based on its understanding of the context, ChatGPT uses its knowledge base and language patterns to construct replies and generate coherent and context-relevant responses.
4. Iterative interaction: in continuous dialogue, ChatGPT can retain dialogue history and context, enabling it to use previous interactions to generate more appropriate responses and ensure smooth dialogue.
5. Output delivery: ChatGPT presents the generated response to the user in human-readable text to complete the dialogue.

Due to its advanced transformer model, ChatGPT excels at understanding and generating language. This capability expands its application potential across diverse fields, notably enhancing interactions and decision support. In medicine, it promises improvements in patient care, telehealth, information retrieval, and education, etc [11-13].

## Application of ChatGPT in Hospital Pharmacy

### Daily Operations in Hospital Pharmacy

#### Drug Information Retrieval and Interpretation

In daily hospital pharmacy operations, pharmacists often need to quickly and accurately retrieve the latest pharmaceutical information. ChatGPT could be used as an auxiliary tool for drug information retrieval and interpretation [14]. Pharmacists can enter specific information for querying, such as drug name, indication, contraindication, medication regimen, adverse reactions, clinical guideline, etc, and provide relevant information [15], thus helping pharmacists save time on information retrieval, improve learning efficiency, and keep their pharmaceutical knowledge up-to-date [16]. In a recent study assessing ChatGPT’s proficiency in clinical pharmacy knowledge, the AI language model was presented with 264 multiple-choice questions commonly used to maintain clinical pharmacists’ basic knowledge. ChatGPT not only achieved a higher accuracy rate of 79% in answering these questions but also demonstrated excellent reproducibility and quality of substantiation, surpassing the performance of pharmacists by a notable margin [17].

#### Prescription Review and Medication Guidance

ChatGPT can assist pharmacists in the review of prescriptions and offer guidance for the rational use of medications. When used for prescription review, ChatGPT can interact with prescribers, feedback-inappropriate prescriptions or medication orders, and improve the work efficiency of prescription review. For controversial prescriptions or medication orders, they are then submitted to clinical pharmacists for manual review. In terms of medication guidance, pharmacists can enter relevant information of specific patients, such as medical history, laboratory test results and comorbidities, and seek advice from ChatGPT on the most appropriate drug therapy regimen for the patient. By considering the patient’s individual factors, drug information, and evidence-based guidelines and other comprehensive factors, ChatGPT supports pharmacists in making decisions regarding drug selection, medication regimen adjustment, and individualized treatment [18]. Finally, pharmacists integrate the relevant suggestions with their own professional medical knowledge and provide individualized medication guidance and optimized medication regimen for patients [15]. The potential of ChatGPT in proactive polypharmacy management was explored. By processing clinical scenarios, ChatGPT demonstrated an ability to make deprescribing decisions, suggesting it can assist in reviewing prescriptions and guiding medication use. The findings indicate that with specialized training, ChatGPT could offer valuable clinical assistance to primary care physicians in managing complex medication regimens for older patients [19].

#### Drug Interaction Management

For patients with multiple medications, managing drug interactions is important and could reduce the potential risks. Upon receiving detailed clinical information and medication profiles from patients, an in-depth analysis can be conducted to identify and prevent possible drug interactions. By accessing specialized databases, a comprehensive evaluation of these drug interactions can be carried out, providing professional advice to patients on how to avoid harmful interactions [15]. This helps pharmacists optimize medication regimens, minimize the risk of adverse reactions, and improve the safety of patient medication. There have been reports that ChatGPT has achieved 95% accuracy in predicting and explaining drug-drug interactions. It can generate logical and coherent text output, rather than simply listing the types and mechanisms of interactions [20]. The application of ChatGPT in medication therapy management has the potential to enhance patient safety and involvement, lower health care costs, and assist health care providers in medication management and identifying drug interactions [21]. In a comparative analysis of AI chatbots’ ability to predict drug-drug interactions, Bing AI outperformed others, including different versions of ChatGPT. Despite some limitations, these AI tools showed promise, with ChatGPT demonstrating potential in accurately identifying drug-drug interactions. This underscores the budding utility of AI, and specifically ChatGPT, in streamlining drug interaction management to bolster patient safety [22].

#### Medication Education and Consultation

Effective medication education and consultation are essential for encouraging patients to actively participate in drug therapy, which is important for promoting the safe and effective use of drugs, improving medication adherence, and optimizing treatment outcomes. However, the reality is that clinical pharmacists have limited time and resources, which limited the extent of medication education and consultation they can provide. ChatGPT could be used as a virtual resource for patient medication education [23], providing information on drug indications, usage, dosage, potential adverse effects, precautions, etc [24]. By serving as a digital assistant, ChatGPT can support pharmacists in consulting on information related to drugs and diseases [25]. It can answer common questions raised by patients and provide professional explanations, such as when to take medicine, how many times a day, what dose to take, whether some foods or alcohol have any effect on medicine, etc, to help patients reduce the risk of medication errors.

**Figure 2:**
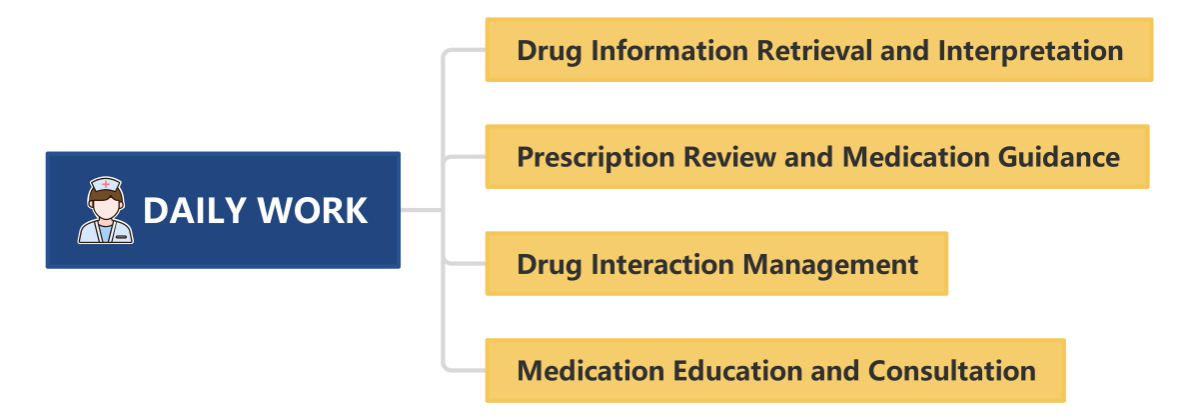
Summary of the application of ChatGPT in daily work in hospital pharmacy.

ChatGPT’s language processing ability is outstanding, which can solve the language barrier problem in the process of medication education and consultation. ChatGPT’s multilingual support ensures comprehensive medication guidance for patients, regardless of their language preferences [26]. A real-world study found that ChatGPT exhibited a higher appropriateness rate in responding to public medication consultation questions compared to those asked by health care providers in a hospital setting [27]. It is particularly important to note that the medication education and consultation services provided by ChatGPT need to be reviewed by pharmacists to ensure the accuracy of information [28]. Pharmacists support the use of ChatGPT in pharmacy practice but have concerns about its use due to ethical reasons, legal problems, privacy concerns, worries about the accuracy of the data generated, data learning, and bias risk [29]. A summary of the application of ChatGPT in daily work of hospital pharmacy is provided in Figure 2.

### Application of ChatGPT in Research

#### Literature Review and Analysis

ChatGPT serves as a tool for pharmacists delving into literature, as it aids in the reviews and analyses process [14]. When presented with specific research topics or questions by researchers, it facilitates the retrieval of relevant literature and synthesizes findings, innovations, future trends, and current research gaps. With ChatGPT’s NLP ability, researchers can accelerate the screening, learning, and summarization of related literature and relevant knowledge and development trends in specific fields. For nondomain knowledge, it is possible to sort out relevant knowledge points through progressive questioning, quickly understand the field, and build a knowledge system. By summarizing and abstracting in a concise way, it provides a way for researchers to quickly understand the research achievements of predecessors, saving time for consulting a large volume of literature. In a systematic review assessing medication adherence improvement in patients with ischemic stroke via mobile health interventions, ChatGPT was pitted against human researchers for identifying relevant studies. While human researchers showed a slightly higher precision (0.86) and relevance percentage (9.8%), ChatGPT demonstrated significant efficiency by identifying a substantial number of relevant studies, including a majority of studies that reported adherence improvements, in a fraction of the time required by human researchers. This case illustrates the utility of ChatGPT in literature review and analysis, suggesting that its speed and broad search capabilities can augment traditional research methods, particularly in the initial stages of study identification [30].

#### Research Topic Selection and Research Design

Researchers can engage in role-playing to consult ChatGPT on a range of questions related to specific field-related research topics, including representative literature, study design, inclusion and exclusion criteria, sample size calculation, research methods, quality control, etc. By offering expert-level insights, ChatGPT serves as a reference for researchers [31]. With ChatGPT’s guidance, researchers not only enrich the research process but also enhance the overall integrity and impact of the scholarly work produced. A study explores the utility of AI-based transformers, specifically ChatGPT, in aiding epidemiological research. By converting STROBE (Strengthening the Reporting of Observational Studies in Epidemiology) guidelines into prompts, ChatGPT was assessed on its ability to generate coherent and relevant responses for observational studies. With mean scores of 3.6 for coherence and 3.3 for relevance, ChatGPT proved to be a valuable asset, particularly in adhering to recognized research standards. However, the study underscores the need for a critical approach to AI-generated content, highlighting the importance of user expertise and awareness of ethical considerations in scientific research [31].

#### Data Statistical Analysis and Plotting

ChatGPT offers a robust suite of support for data statistical analysis, enabling researchers to extract meaningful information from datasets. ChatGPT’s Code Interpreter plugin is a powerful tool that can translate natural language into executable code for data statistical analysis and plotting. After entering datasets or specific research questions, ChatGPT suggests suitable statistical techniques and interprets the data accordingly. ChatGPT is adept at generating code in programming languages such as Python, SAS, R, etc, and then using Python and other tools for data mining, analysis, and plotting [32]. ChatGPT can work with interactive plotting software to assist researchers in generating various types of figures, including scatter plots, bar charts, line charts, heat maps, and network graphs. By understanding researchers’ input or description of data, ChatGPT could offer plotting suggestions based on the research objectives of the data, recommending the most appropriate graphic types, layouts, and color schemes that best represent the information conveyed. One study compared the statistical analysis capabilities of ChatGPT-4 with traditional biostatistical software (SAS, SPSS, and R) using data from the China Health and Nutrition Survey. ChatGPT-4 demonstrated high consistency, efficiency, and user-friendliness in descriptive statistics, and maintained these advantages in intergroup and correlational analyses despite minor discrepancies with the traditional software. ChatGPT-4 is a powerful tool for epidemiological data analysis, particularly for researchers with intermediate data analysis experience. It suggests that AI’s integration with data analysis platforms can simplify operations and enhance research focus on result interpretation, potentially revolutionizing epidemiological and medical research [33].

#### Academic Writing

**Figure 3:**
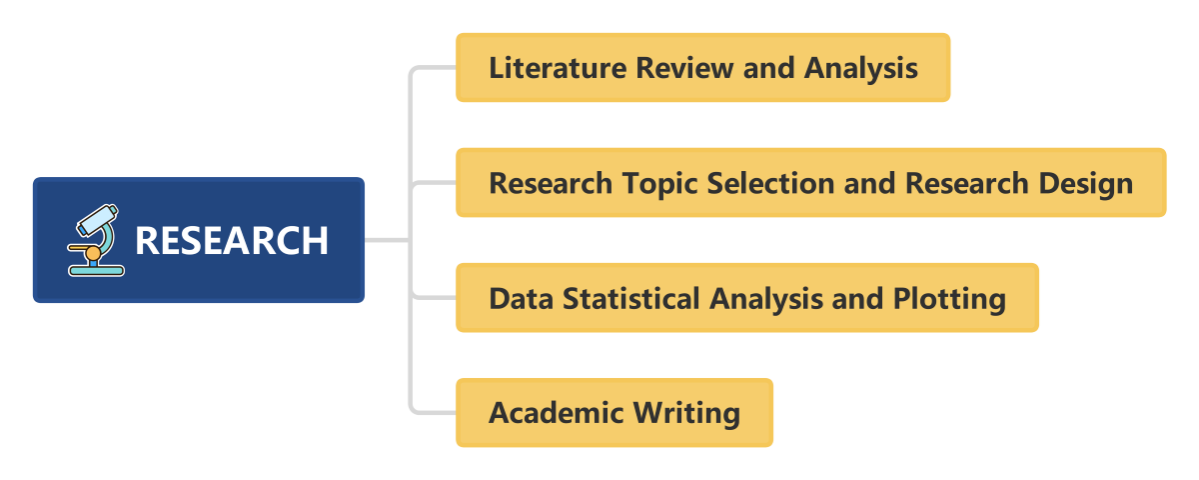
Comprehensive artificial intelligence–enhanced scientific research framework incorporating literature review, topic selection, design, data analysis, and result interpretation.

ChatGPT has a very obvious advantage in assisting paper writing [34]. It offers strategic advice on the structure of the paper, guiding researchers in the logical arrangement of the sections and content. In academic paper writing, the accuracy and fluency of language expression are very important. ChatGPT aids in polishing the manuscript’s language and improving the paper’s quality and readability [28,35,36]. However, it should be noted that although ChatGPT can provide a lot of help in paper writing, there is the potential for misunderstandings or errors [37]. It should not supplant the professional knowledge and judgment of researchers [38,39]. Therefore, when using ChatGPT, researchers should verify the reliability of its output results [40]. Simultaneously, ChatGPT should not be used to write entire papers, but rather be used as an auxiliary tool to assist researchers in research and writing [41,42]. ChatGPT serves as a versatile technological tool, one whose influence can be profoundly positive or negative, depending on the wisdom and discretion with which it is used. It holds significant promise as an asset to scientific writing; yet, it lacks the ability to supersede the irreplaceable qualities of human composition and the nuanced faculties of critical thought. Hence, the use of AI in academic writing should be conducted with unwavering adherence to the foundational principles of honesty, rigor, and originality [43]. A summary of the application of ChatGPT in research is provided in Figure 3.

## Challenges and Issues Faced by ChatGPT

Although ChatGPT enhances the efficiency of daily work in hospital pharmacy and provides assistance in scientific research, it still faces some technical hurdles and challenges [28,44-46]. ChatGPT’s responses rely on the context provided in the input. However, it may sometimes misunderstand or misinterpret vague queries or complex medical scenarios, resulting in potentially inaccurate or irrelevant responses. Repeated questions to ChatGPT do not ensure consistent answers [24]. Its knowledge is rooted in pretraining data, which means that it lacks the ability to update medical guidelines, latest research results, or constantly evolving clinical practices in real time. Therefore, its answers may not be the most up-to-date information [31]. In addition, biases present in its training data may influence its responses, introducing the risk of biased or incorrect content [47,48]. The performance of ChatGPT-4 and Google Bard in providing accurate preventive medicine and primary care recommendations were evaluated, and a considerable portion of responses from both AI models contained inaccuracies or missing details. The findings underscore the necessity for AI tools to be regularly updated, especially in fast-paced medical fields, and to be used as supplementary resources rather than sole authorities on medical information [49].

**Figure 4:**
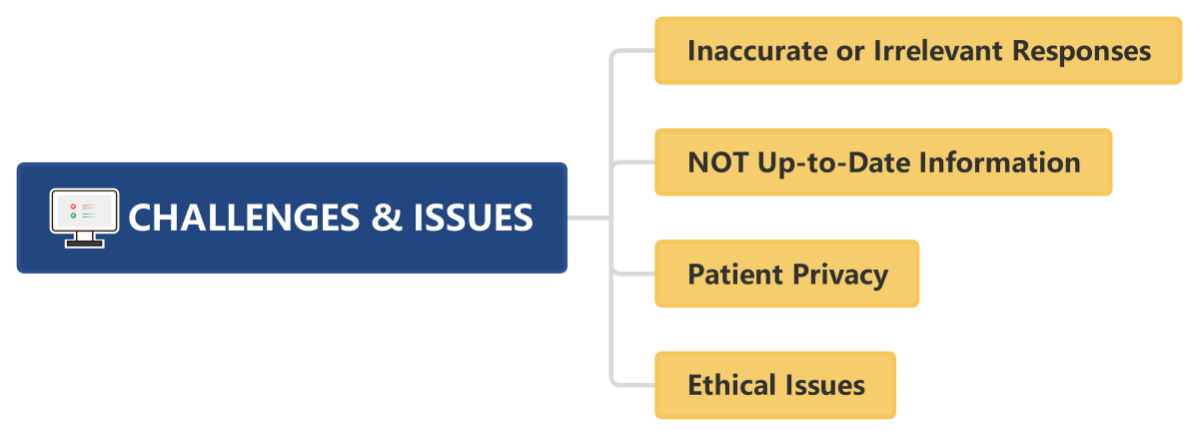
Key challenges and issues in artificial intelligence applications highlighting inaccurate responses, outdated information, patient privacy, and ethical concerns.

Incorporating ChatGPT into hospital settings requires a careful approach to patient privacy and ethical considerations [50]. When interacting with ChatGPT, sensitive information should be deidentified to protect patient privacy. In daily work and scientific research, one should be clear about the advantages and disadvantages of using ChatGPT and obtain informed consent from patients before using ChatGPT. ChatGPT, while capable of addressing professional inquiries and providing recommendations, serves as an auxiliary decision support tool rather than a substitute for the professional knowledge of clinical pharmacists [51]. Pharmacists or researchers should critically evaluate and verify the information provided by ChatGPT before making a final decision, and they should be responsible for the final decision (Figure 4).

## Thoughts on AI Development

While the potential of ChatGPT in enhancing hospital pharmacy operations is significant, the practicalities of its integration into existing workflows require careful consideration. The necessary infrastructure includes robust IT systems capable of handling the computational demands of AI, secure data storage solutions to safeguard patient privacy, and a reliable internet connection to ensure continuous access to ChatGPT’s services. Staff training is equally vital, encompassing not only the technical aspects of using the AI tool but also the interpretation of its outputs within a clinical context. Change management strategies must be used to facilitate a smooth transition, addressing potential resistance and fostering a culture of continuous learning and adaptation. Piloting ChatGPT in select areas of the pharmacy, soliciting feedback from frontline staff, and iterating on the implementation plan based on real-world experiences can significantly mitigate challenges and optimize outcomes. Ongoing evaluation and refinement of the integration process are imperative to ensure that the benefits of ChatGPT are fully realized and sustained over time.

To facilitate the development and scientific rational use of AI, it is imperative for governments and industry associations to establish pertinent innovation and regulatory frameworks [37,50]. Innovation policies include formulating technical standards for AI, supporting the research and development of AI, increasing investment in AI technology, strengthening talent cultivation for AI, promoting popularization and promotion of AI, etc. Regulatory policies include strengthening protection of intellectual property rights related to AI, ensuring that AI is only used for reasonable and legal activities, ensuring that relevant policies are conducive to innovation and development of AI, protection of privacy by AI, ethical issues related to AI, etc. The United States has already formulated relevant policies in this regard.

To optimize ChatGPT’s role in hospital pharmacy, enhancing its model performance and reliability is important [26]. On the basis of the original data model, it is crucial to strengthen its training on professional medical datasets. This will enhance its understanding of medical terminology, drug information, and clinical background knowledge. Refining its ability to deliver precise responses to medical inquiries is paramount. By bolstering ChatGPT's interpretive skills in grasping nuanced contexts, it can generate more accurate and contextually fitting answers. Advancing its contextual understanding will not only minimize the incidence of incorrect responses but also elevate its efficacy in intricate medical situations. Medical professionals should participate in training and calibration of ChatGPT and regularly evaluate and verify its performance in medical applications. Through rigorous testing, comparison with gold standards for calibration, and incorporation of feedback from medical professionals, its reliability in the medical field can be enhanced.

**Figure 5:**
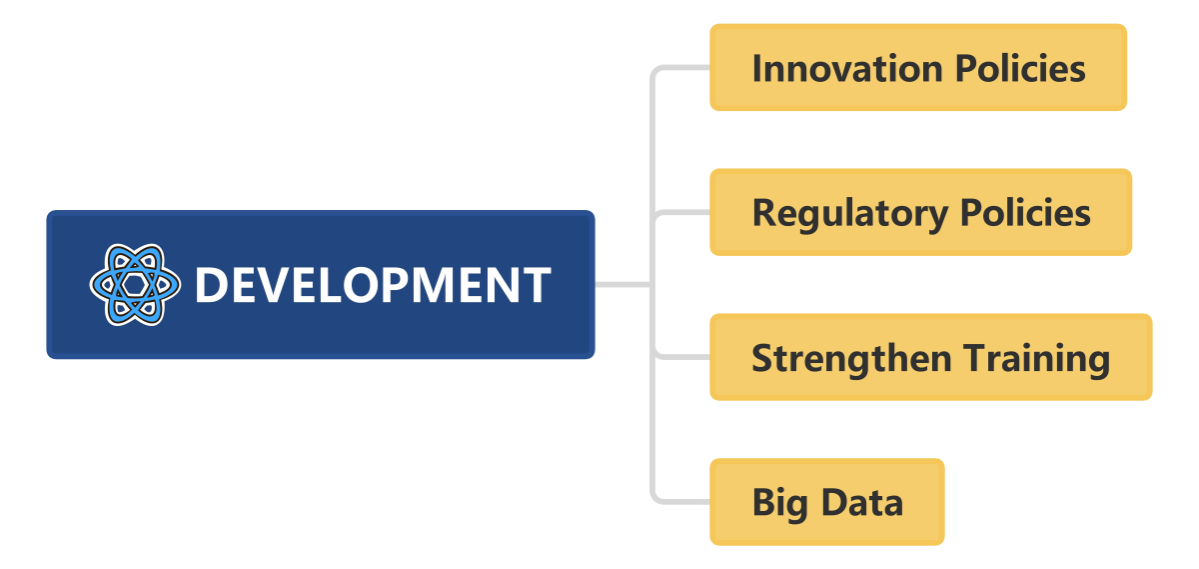
Strategic recommendations for the future development of artificial intelligence.

AI-assisted diagnosis is based on the intelligent analysis of big data. It is necessary to solve the problem of fragmentation of medical health data to achieve a leap from data to knowledge to intelligence. Efforts must be made to bridge the gaps among disparate data sources, creating a comprehensive medical knowledge center that facilitates a connection between individuals and health care providers. To ensure the widespread adoption of AI in health care, developing interfaces that seamlessly integrate AI technologies with existing hospital information systems is essential. One study introduces PMC-LLaMA, an open-source large language model tailored for medical applications. By integrating a vast database of biomedical literature and textbooks, and fine-tuning with domain-specific instructions, PMC-LLaMA has reportedly outperformed models like ChatGPT in medical question–answering benchmarks. The research provides an open-source framework that could drive future developments in medical AI, offering a foundational model that can be further trained and adapted for various medical tasks [52]. This integration will enable AI to play role in enhancing hospitals’ information infrastructure (Figure 5).

Integrating ChatGPT into the practice of hospital pharmacy requires a detailed strategy that addresses technology, training, data security, and ethical compliance across several key areas. Initially, a needs assessment determines the scope of assistance that ChatGPT can provide, such as drug information retrieval and patient counseling. Subsequently, IT infrastructure is strengthened to ensure that servers, data storage, and network security can support the integration of ChatGPT, with the development of data interfaces for seamless integration with hospital systems like electronic health records. Concurrently, pharmacy personnel are trained in the use of ChatGPT, emphasizing best practices and potential risks. In terms of privacy and compliance, it is imperative that the use of ChatGPT adheres to data protection regulations such as HIPAA (Health Insurance Portability and Accountability Act) and undergoes review by an ethics committee to prevent ethical issues. Moreover, strict data security protocols and oversight mechanisms are implemented to protect patient information and monitor ChatGPT’s performance. Intuitive user interfaces have been designed to facilitate interaction between medical staff and ChatGPT, and testing in real environments has been conducted, with feedback collected for iterative improvements. Regular assessments of ChatGPT’s performance, including accuracy and user satisfaction, are conducted, and necessary improvements are made based on the evaluation results. Medical personnel are encouraged to engage in research on the application of ChatGPT in pharmacy and to publish their findings to share knowledge. Patients are clearly informed about the supportive role of ChatGPT, its benefits, and the data security measures in place, maintaining full transparency. Additionally, collaboration across various disciplines such as pharmacy, IT, data science, and health care management is fostered to ensure the effective integration of ChatGPT. Finally, clear policies and guidelines for AI use are established to ensure that all relevant personnel understand and comply with these regulations. Through these comprehensive strategies, hospital pharmacy departments can improve work efficiency while ensuring patient safety and data protection.

## Conclusion

In summary, with its advanced language generation and understanding capabilities, ChatGPT has great value and broad application prospects in the field of hospital pharmacy. Its potential to streamline daily tasks and spur advancements in scientific research is noteworthy. However, there are still some challenges with ChatGPT’s predictive performance, privacy protection, and ethics. In the future, through policy formulation, model optimization, information integration, and other measures are required to further promote the development and application of AI in hospital pharmacy. ChatGPT is expected to contribute to the transformation of hospital pharmacy in the future, benefiting patients, pharmacists, medical practitioners, and the broader health care ecosystem.

## Abbreviations

AI: artificial intelligence
GPT: generative pretrained transformer
HIPAA: Health Insurance Portability and Accountability Act
NLP: natural language processing
STROBE: Strengthening the Reporting of Observational Studies in Epidemiology
